# PARP-1-dependent RND1 transcription induced by topoisomerase I cleavage complexes confers cellular resistance to camptothecin

**DOI:** 10.1038/s41419-018-0981-3

**Published:** 2018-09-12

**Authors:** Laetitia Mouly, Kenza Mamouni, Remi Gence, Agnese Cristini, Julia Cherier, Adrien Castinel, Morgane Legrand, Gilles Favre, Olivier Sordet, Sylvie Monferran

**Affiliations:** 1 Cancer Research Center of Toulouse (CRCT), INSERM, Université de Toulouse, Université Toulouse III Paul Sabatier, CNRS, Toulouse, France; 2 Faculté des Sciences Pharmaceutiques, Université de Toulouse, Université Toulouse III Paul Sabatier, Toulouse, France

## Abstract

RHO GTPases regulate essential functions such as the organization of the actin cytoskeleton. The classic members cycle between an active GTP-bound and an inactive GDP-bound conformation whereas atypical members are predominantly GTP-bound. Besides their well-established role, the classic RHO GTPases RHOB and RAC1, are rapidly induced and/or activated by genotoxic stress and contribute to the DNA damage response. Here we used camptothecin, a selective topoisomerase I (TOP1) inhibitor that stabilizes TOP1 cleavage complexes (TOP1cc), to search for other potential early DNA damage-inducible RHO GTPase genes. We identified that an atypical RHO GTPase, *RND1*, is rapidly induced by camptothecin. *RND1* induction is closely associated with the presence of TOP1cc induced by camptothecin or by DNA lesions that elevate TOP1cc levels such as UV and hydrogen peroxide. We further demonstrated that camptothecin increases *RND1* gene transcription and mRNA stability. Camptothecin also increases poly(ADP-ribose) polymerase 1 (PARP-1) activity, whose inhibition reduces *RND1* transcription. In addition, overexpression of RND1 increases PARP-1, suggesting a cross-talk between PARP-1 and RND1. Finally, RND1 protects cells against camptothecin-induced apoptosis, and hence favors cellular resistance to camptothecin. Together, these findings highlight RND1 as an atypical RHO GTPase early induced by TOP1cc, and show that the TOP1cc-PARP-1-RND1 pathway protects cells against apoptosis induced by camptothecin.

## Introduction

The RHO GTPase family comprises 20 members in human, which can be divided into classic and atypical members^1^. Classic RHO GTPases, such as RHOB and RAC1, cycle between an active GTP-bound and an inactive GDP-bound conformation. Atypical RHO GTPases, such as RND1, are unable to hydrolyze GTP and are therefore in a constitutive active GTP-bound conformation^2,3^. Other atypical members, such as RHOU, and presumably also RHOV, have a high nucleotide exchange rate and hence are assumed to be mainly GTP-bound^4^. Consequently, the tight control of the expression of atypical RHO GTPases is important to precisely tune their activity. GTP-bound RHO GTPases bind to their effectors and regulate pivotal cellular functions, including the organization of the actin and microtubule cytoskeletons, cell adhesion and cell migration^5^.

Besides their canonical roles, the RHO GTPases RAC1 and RHOB have been implicated in the early response to DNA damage. Inhibition or deletion of RAC1 reduces the DNA damage signaling pathway upon UV light^6^ or ionizing radiation^7^ and, sensitizes cell to ionizing radiation^7^ or to UV-light-induced apoptosis^6^. Unlike RAC1 that is primarily activated in response to DNA damage without change in expression^7,8^, RHOB is both induced and activated^9–12^. RHOB induction by genotoxic stress, such as UV light and the topoisomerase I (TOP1) inhibitor camptothecin (CPT), is rapid and relies on increased transcription and/or transcript stability^9,10^. Increased expression of RHOB promotes DNA repair and confers cell resistance to genotoxic stress^9^. At present, it is not known whether, besides RHOB, other RHO GTPases are early DNA damage-inducible genes, at the expression level.

TOP1 solves DNA topological problems that are generated during transcription and replication^13^. It relaxes DNA by forming transient TOP1 cleavage complexes (TOP1cc), which are TOP1-linked DNA single-strand breaks . After DNA relaxation, TOP1cc reverse rapidly, and TOP1 is released as the DNA religates. The transient TOP1cc can be trapped selectively by CPT and its derivatives irinotecan and topotecan, used to treat cancers, which bind at the TOP1-DNA interface^14^. Many DNA alterations including oxidative base damages^15,16^ and UV lesions^17,18^ also interfere with TOP1 nicking-closing reactions and give rise to elevated levels of TOP1cc (see Table 1 in ref. ^13^). Persistent TOP1cc can lead to the production of DNA double-strand breaks (DSBs) during replication^19–21^ and transcription^22–24^, and ultimately to apoptotic cell death^25^.

An early response to long-lived TOP1cc is the interference with the progression of transcription^14,26^. Indeed, trapping TOP1cc by CPT inhibits transcription elongation with increasing efficiency as the genes become longer and contain more exons^27–29^. However, genes are differentially affected by CPT and a fraction of them, primarily the short and low-expressed genes, are upregulated^27,28^. The mechanisms by which CPT-induced TOP1cc trapping enhances transcription at some genes are largely unknown. Here, we identified RND1 as the first atypical RHO GTPase, which is rapidly induced at the gene level by CPT and DNA damaging agents that indirectly trap TOP1cc, such as hydrogen peroxide (H_2_O_2_) and UV light. We found that persistent TOP1cc increase RND1 transcription by a mechanism that depends on poly(ADP-ribose) polymerase 1 (PARP-1) activity, providing one of the first examples of how stabilized TOP1cc can stimulate gene transcription. Lastly, we found that increased RND1 expression reduces CPT-induced apoptosis, highlighting a protective function for the TOP1cc-PARP-1-RND1 pathway.

## Material and Methods

### Drugs, chemical reagents

CPT, H_2_O_2_, flavopiridol (FLV), actinomycin D, cobalt(II) chloride (CoCl_2_), paclitaxel, methotrexate (MTX), 5-aza-2-deoxycytydine (5AZA), trichostatin A (TSA), and the ATR inhibitor VE-821 were obtained from Sigma-Aldrich, the PARP inhibitor veliparib and the DNA-PK inhibitor NU7441 from Selleckchem, and the ATM inhibitor KU55933 from Millipore. H_2_O_2_, CoCl_2_ and actinomycin D were dissolved in water, MTX in 0.1 M sodium hydroxide and the other agents in DMSO.

### Cell lines, culture and treatments

Human osteosarcoma (U2OS), glioblastoma (U87), and colon carcinoma (HCT116) cells, and murine melanoma (B16F10), and embryonic (NIH3T3) cells were obtained from the American Type Culture Collection (ATCC), and cultured in Dulbecco’s modified Eagle’s medium (DMEM) supplemented with 10% (v/v) fetal bovine serum. HCT116 cells of each genotype (p53+/+ and p53−/−) were kind gifts from Dr. Bert Vogelstein (John Hopkins Kimmel Cancer Center, Baltimore, MD). Primary human lung embryonic WI38 fibroblasts immortalized with hTERT were obtained from Estelle Nicolas (LBCMCP, Toulouse, France) and Carl Mann (CEA, Gif-sur-Yvette, France)^30^ and cultured in modified Eagle’s medium (MEM) supplemented with 10% (v/v) fetal bovine serum, 1 mM sodium pyruvate, 2 mM glutamine and 0.1 mM non-essential amino acids. In Fig. 2a, cells were irradiated at 500 J/m^2^ with a UVB lamp RMX3W system (312 nm) from BioSun (Vilber Lourmat). In all the experiments, mock samples were only treated with the vehicle.

### Cell transfection and transduction

To establish U2OS shRND1 and shCtrl cell lines, 3,000 U2OS cells were transfected using jetPEI reagent (Polyplus), with 1 µg of shRNA plasmid with a sequence directed against *RND1* mRNA (5′-GGACAGAAATCCTAGATTATT-3'; QIAGEN) or a control sequence (5′-GGAATCTCATTCGATGCATAC-3′; QIAGEN). 2days after transfection, transfected cells were seeded at low density and treated with puromycin. After 2 to 4 weeks of selection, resistant clonal cells appeared, were removed with cloning cylinder and then amplified.

For transient siRNA transfection in Fig. 2d, U2OS or WI38 hTERT cells were transfected for 48 h with 50 nM of siRNA duplexes against TOP1 (5′-GGACUCCAUCAGAUACUAUdTdT-3′; QIAGEN) or a non-targeting sequence (SR-CL000–005; Eurogentec) with Dharmafect 4 transfection reagent (GE Healthcare).

For transient transfection in Fig. 7e, 1.5 million U2OS cells were transfected with 10 µg of p-EGFP-RND1 (Addgene) or with 10 µg of p-EGFP (Clontech) using jetPEI reagent (Polyplus) according to the manufacturer’s protocol. Forty-eight hours after transfection, cells were sorted by FACS in either GFP-positive or GFP-negative RND1.

To establish U2OS RND1-V5 cell lines, 20,000 (for RND1-V5-high) or 40,000 (for RND1-V5-low) U2OS cells were transduced in complete medium with 10 µg/mL of polybrene with lentiviral particles (MOI of 5:1) containing the pLX317-puromycin-RND1-V5 (which contains the cDNA of *RND1*; Sigma-Aldrich) or a control sequence (tGFP; Sigma-Aldrich). 3 days after transduction, cells were selected with puromycin.

### Quantitative reverse transcription-PCR

**Fig. 1:**
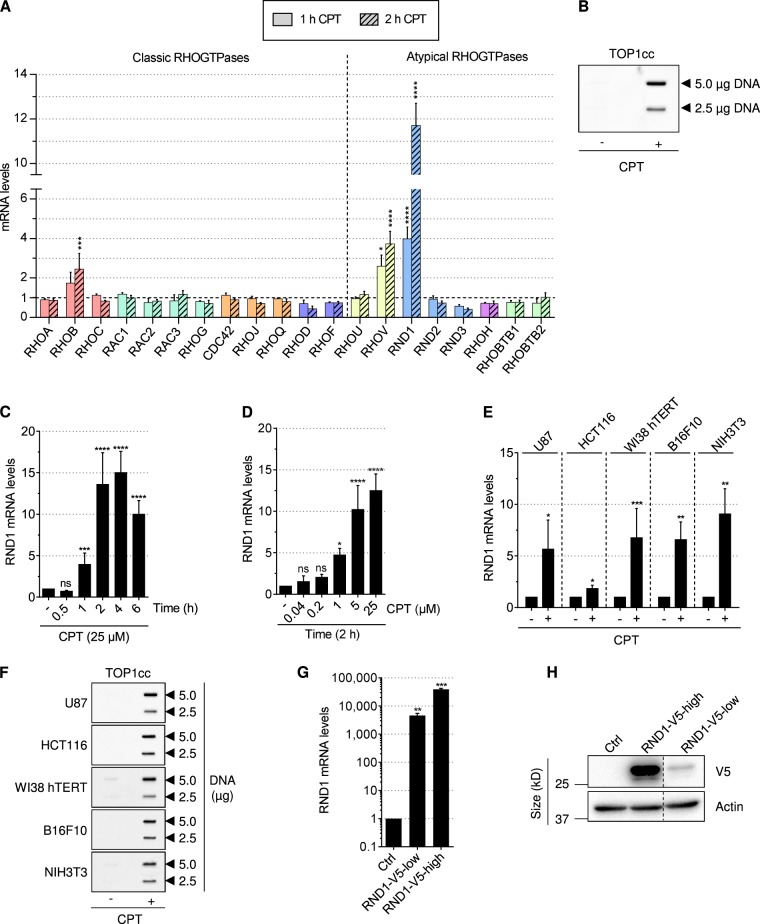
Rapid induction of *RND1* transcripts by CPT. **a** RT-qPCR analysis of *RHO GTPase* mRNA in U2OS cells treated with 25 μM CPT for the indicated times. Data were normalized to that of untreated cells, which was denoted by the dashed line (means ± SEM, *n* ≥ 3). *P < 0.05, ***P < 0.001, ****P < 0.0001 by two-way ANOVA. Colors are RHO GTPase sub-families. **b** Detection of TOP1cc in U2OS cells treated with 25 µM CPT for 1 h. Two concentrations of genomic DNA (5 and 2.5 µg) were probed with an anti-TOP1cc antibody. **c**, **d** RT-qPCR analysis of *RND1* mRNA in U2OS cells treated for the indicated times with 25 µM CPT (**c**), and with the indicated CPT concentrations for 2 h (**d**). The data are expressed as means ± SD for *n* ≥ 3, *P < 0.05, ***P < 0.001, ****P < 0.0001 by one-way ANOVA. **e**, **f** Cells were treated with 25 µM CPT for 2 h (U87, HCT116, and WI38 hTERT cells) or 4 h (B16F10 and NIH3T3 cells). (**e**) RT-qPCR analysis of *RND1* mRNA (means ± SD, *n* ≥ 3). *P < 0.05, **P < 0.01, ***P < 0.001 by unpaired *t* test. **f** Detection of TOP1cc. Two concentrations of genomic DNA (5 and 2.5 µg) were probed with an anti-TOP1cc antibody. **g**, **h** U2OS were stably expressing V5-tagged RND1 at low (RND1-V5-low) or high levels (RND1-V5-high) or EGFP (ctrl). **g** RT-qPCR analysis of *RND1* mRNA (means ± SD, *n* = 3). **P < 0.01, ***P < 0.001 by unpaired *t* test. **h** Western blotting analysis of V5 tag. Actin: loading control. Dashed lines indicate that intervening wells have been spliced out. Ns, not significant.

**Fig. 2:**
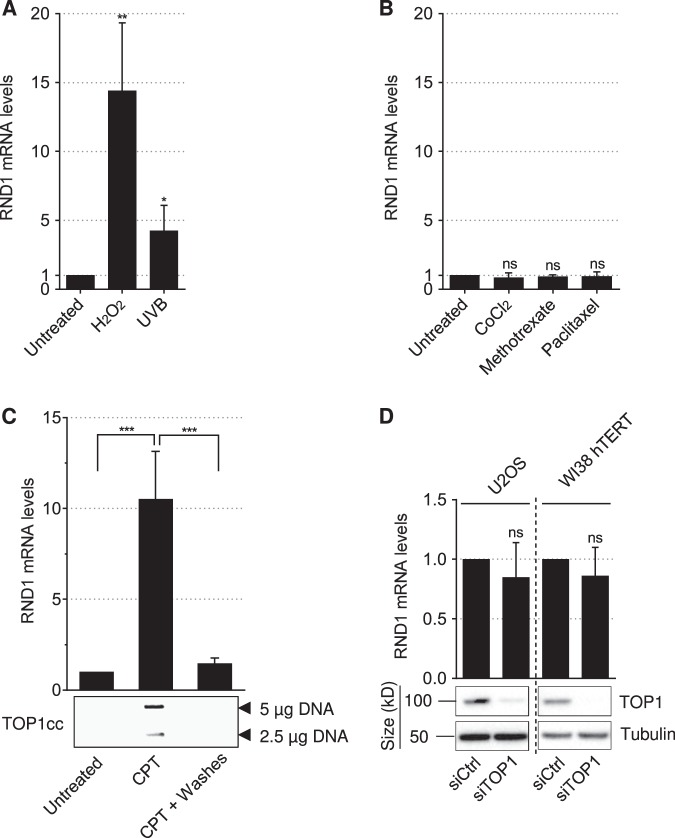
*RND1* transcripts are closely associated with the presence of TOP1cc. **a**, **b** RT-qPCR analysis of *RND1* mRNA in U2OS cells treated for 2 h with 1 mM H_2_O_2_ or irradiated with 500 J/m^2^ UVB (**a**), or treated with 100 µM CoCl_2_, 50 µM methotrexate or 10 µM paclitaxel (**b**). Treatment with CPT has been performed in parallel as a positive control (not shown). Data are expressed as means ± SD for *n* = 3, *P < 0.05, **P < 0.01 by unpaired *t* test. **c** U2OS cells were treated with 25 µM CPT for 2 h and washed and cultured in CPT-free medium (CPT + Washes) for 2 h to allow reversion of TOP1cc. Top panel: *RND1* mRNA was analyzed by RT-qPCR (means ± SD, *n* = 3). ***P < 0.001 by one-way ANOVA. Bottom panel: Detection of TOP1cc. Two concentrations of genomic DNA (5 and 2.5 µg) were probed with an anti-TOP1 antibody. **d** U2OS and WI38 hTERT cells were transfected with siRNAs against TOP1 (siTOP1) or against a control sequence (siCtrl). Bottom panel: efficiency of the siRNA determined by Western blot. Tubulin: loading control. Top panel: *RND1* mRNA was analyzed by RT-qPCR (means ± SD, *n* = 3). Ns, not significant.

Total RNAs were extracted using the RNeasy Plus mini kit (QIAGEN) according to the manufacturer’s instructions and the concentration and purity of RNA were determined using the Nanodrop ND-1000. RNAs were reverse transcribed using the iScript cDNA synthesis kit (Bio-Rad). qPCR analyses were performed on a CFX96 real-time system device (Bio-Rad) by using IQ SYBR green Supermix (Bio-Rad) according to the manufacturer’s instructions. All samples were analyzed in triplicate, and actin, GAPDH and 28 S mRNA were used as endogenous controls in the ΔΔCT analysis. The human (*h*) and mouse (*m*) primer pairs used were *hRHOA*-FW (5′-TGGAAGATGGCATAACCTGTC-3′) and *hRHOA*-RV (5′-AACTGGTGGCTCCTCTGG-3′), *hRHOB*-FW (5′-TTGTGCCTGTCCTAGAAGTG-3′) and *hRHOB*-RV (5′-CAAGTGTGGTCAGAATGCTAC-3′), *hRHOC*-FW (5′-TGTCATCCTCATGTGCTTCTC-3′) and *hRHOC*-RV (5′-GTGCTCGTCTTGCCTCAG-3′), *hRAC*1-FW (5′-AGAACACCGAGCACTGAAC-3′) and *hRAC1*-RV (5′-ACGCATCTGAGAACTACATAGG-3′), hRAC2-FW (5′-GGACAGCAAGCCAGTGAAC-3′) and hRAC2-RV (5′-GGAGAAGCAGATGAGGAAGAC-3’), hRAC3-FW (5’-GTGATGGTGGACGGGAAAC-3′) and hRAC3-RV (5′-CACTTGGCACGAACATTCTC-3′), *hRHOG*-FW (5′-CCGCTCTCACTTCCTTCTC-3′) and *hRHOG*-RV (5′-ACCACCACGCACTTGATG-3′), *hCDC42*-FW (5’-GTCAAGTATGTGGAGTGTTCTG-3′) and *hCDC42*-RV (5′-CACCTGCGGCTCTTCTTC-3′), *hRHOJ* (QIAGEN; QT00092078), *hRHOQ*-FW (5′-TATGCCAACGACGCCTTC-3′) and *hRHOQ*-RV (5′-GCCGTGTCA TAGAGTCCTAG-3′), *hRHOD*-FW (5′-GATTGGAGCCTGTGACCTAC-3′) and *hRHOD*-RV (5′-GTAATCCGCCGCCAGAAG-3′), *hRHOF*-FW (5′-CAGACAGACCTCACGACAG-3′) and *hRHOF*-RV (5′-AGTTCCAGAATGTTCCAAGAG-3′), *hRHOU*-FW (5′-CGGTGGTGTCTGTGGATG-3′) and *hRHOU*-RV (5′-GAAGATGTCTGTGTTGGTGTAG-3′), *hRHOV*-FW (5′-CATAGCAAGTAGTAGGCAGGAG-3′) and *hRHOV*-RV (5′-TCAGAGTGGGCAGTTAGAGG-3′), *hRND1a*-FW (5’-GCAAGTGTTAGCGAAGGA-3′) and *hRND1a*-RV (5′-GCAGAGTGGACGGACA-3′), *hRND1b*-FW (5′-CGCTCTGAACTCATCTCTTC-3′) and *hRND1b*-RV (5′-CCATTCCTGTCTCCTTCCAA-3′), *mRND1*-FW (5′-CAGTTGGGCGCAGAAATCTAC-3′) and *mRND1*-RV (5′-TGGGCTAGACTTGTTCAGACA-3′), *hRND2* (QIAGEN QT00219891), *hRND3*-FW (5′-CCTGCTCCTCTCGCTCTC-3′) and *hRND3*-RV (5′-TCTGGCTGGCTCTTCTCTC-3′), *hRHOH*-FW (5′-TTCACCTCCGAGACCTTCC-3′) and *hRHOH*-RV (5′-GCCACAGAGTAGCACATCAG-3′), *hRHOBTB1*-FW (5′-TGGAGCGTTCTCGGGATGT-3′) and *hRHOBTB1*-RV (5′-CGAAAAACAGAGGACCACAACA-3′), *hRHOBTB2*-FW (5′-CAGCCAGCTTTGACGTGTG-3′) and *hRHOBTB2*-RV (5′-TTGCCCCGTAAGATCCCGT-3′), *actin*-FW (5′-TCCCTGGAGAAGAGCTACGA-3′) and *actin*-RV (5′-AGGAAGGAAGGCTGGAAGAG-3′), *GAPDH*-FW ( 5'-TGCACCACCAACTGCTTAGC-3') and *GAPDH*-RV ( 5'-GGCATGGACTGTGGTCATGAG-3') and *28S*-FW (5′-TCGCTGGGTCTTGGATGT-3′) and *28S*-RV (5′-AGCAGATTGTGACAGACCATTCC-3′). *h*RND1*a* primers were designed at exons 1 and 3 and *h*RND1*b* primers at exon 5. *h*RND1*a* primers were used to perform experiments showed in Fig. 1a, c–e (for U87 and HCT116 cells), Figs. 1g, 2a (for UVB) and Fig. 2b. *h*RND1*b* primers were used to perform all the other RT-qPCR experiments.

### Cell extracts and immunoblotting

Whole cell extracts were obtained by lysing cells in 1% SDS and 10 mM Tris-HCl (pH 7.4) supplemented with protease inhibitors (Sigma-Aldrich) and phosphatase inhibitors (Halt phosphatase inhibitor cocktail; ThermoFisher). Viscosity of the samples was reduced by brief sonication. To detect PAR, cell extracts were performed as described previously^31^. To detect ATM, ATM-pS1981, DNA-PK and DNA-PK-pS2056, cell extracts were prepared as previously described^22^. Proteins were separated by SDS-PAGE and immunoblotted with the following antibodies: anti-actin (MAB1501; Millipore), anti-ATM (ab32420; Abcam), anti-ATM-pS1981 (ab81292; Abcam), anti-cleaved caspase-3 (#9664; Cell Signaling), anti-caspase-9 (#9502; Cell Signaling), anti-Chk1 (sc84081; SantaCruz), anti-Chk1-pS345 (#2348; Cell Signaling), anti-DNA-PK (ab1832; Abcam), anti-DNA-PK-pS2056 (ab18192; Abcam), anti-HIF1α (NB100–449; Novus), anti-PAR (AM80–100UG; Millipore), anti-p53 (#48818; Cell Signaling), anti-p53-pS15 (#9284, Cell Signaling), anti-PARP-1 (#9542; Cell Signaling), anti-TOP1 (ab109374; Abcam), anti-Tubulin (T5168; Sigma-Aldrich), anti-V5 tag (46–0705; Invitrogen). Immunoblotting was revealed by chemiluminescence using ChemiDoc MP System (Bio-Rad). Quantification of protein levels was done with Image Lab software (version 4.1).

### WST-1 cell viability assays

GFP-positive and GFP-negative RND1 sorted cells (Astrios, Beckman) were immediately seeded in triplicate into 96-well microplates at a density of 1,000 cells per well. Twenty-four hours after plating, cells were treated with increasing concentrations of CPT (from 1.6 nM to 25 µM) and cultured for 72 h. The WST-1 reagent (Roche Diagnostics) was then applied for 1 h at 37 °C. The formazan dye was quantified at 450 nm using a plate reader (FLUOstar Optima, BMG Labtech). Data were expressed as the percentage of cell survival (mean ± SD of treated cells normalized to the mean ± SD of untreated cells, which was set to 100%).

### Clonogenic assays

Three hundred U2OS shCtrl or shRND1 cells were treated with increasing concentrations of CPT (from 1.25 nM to 20 nM). Ten days after CPT treatment, cells were fixed with 3.7% paraformaldehyde (Sigma-Aldrich) and stained with 1% crystal violet (Sigma). Colonies containing more than 50 cells were counted.

### Nascent RNA transcripts analysis

Nascent RNAs were labeled and captured using the Click-iT Nascent RNA capture kit (Life Technologies) according to the manufacturer’s instructions. A 2-h 5-ethynyl uridine (EU) pulse at the concentration of 0.2 mM was performed to label nascent RNAs. Three to seven µg of total RNA was used for the Click reaction.

### Luciferase reporter assay

To study the activity of *RND1* promoter, three plasmids were used: pGL3-basal promoterenhancer1*RND1*-lucF (kindly provided by Dr Tan, Tianjin, China), pGL3-basalpromoter*RND1*-lucF and pGL3-promoterenhancer2*RND1*-lucF (kindly provided by Dr Minami, Tokyo, Japan). Using jetPRIME reagent (Polyplus), 60,000 U2OS cells were transiently cotransfected with 2 µg pGL3-*RND1* promoter plasmid and 20 ng of pRL-CMV (Promega). Luciferase activities were measured 24 h after transfection by using the Dual Luciferase assay system (Promega). All data were normalized by Renilla luciferase luminescence derived from the cotransfected pRL-CMV as described previously^32^.

### Meta-analysis of *RND1* mRNA expression

This analysis was performed using the online NextbioResearch tools (http://www.nextbio.com/). We collected *RND1* mRNA expression fold-change after treatment with CPT or derivatives in different cancer cells (OCI-LY3 cells: diffuse large B cell lymphoma; MCF-7: breast cancer cells; PC3: prostate cancer cells; HCT116: colon cancer cells) or tissue (bone marrow from rats) from five gene expression datasets. GEO accession numbers of gene expression datasets in order of appearance: GSE63902^33^; GSE51068^34^; GSE18552^35^; GSE5258^36^ and GSE37352^27^.

### Detection of TOP1 cleavage complexes

Cellular TOP1 cleavage complexes (TOP1cc) were detected as previously^22^, except that immunoblotting was revealed with a mouse anti-TOP1cc from Millipore (MABE1084)^37^ in Figs. 1b, f, 4e and Supplementary Figure 2a.

### Flow cytometry

For sub-G1 analysis, cells were fixed with 70% ethanol, incubated with RNase A (Sigma-Aldrich) and stained with propidium iodide (PI; Molecular probes). The stained cells were analyzed on a BD Accuri C6 flow cytometer (BD Biosciences). Analysis was performed with the BD Accuri C6 flow cytometer software.

### Immunofluorescence

Fifteen thousand U2OS cells were seeded on glass coverslips. After treatment, cells were washed with PBS, fixed in 3.7% paraformaldehyde for 10 min, and permeabilized with 0.2% Triton X-100/1% bovine serum albumin (BSA)/PBS buffer for 5 min. Cells were incubated with 10% BSA for 30 min to block non-specific binding before incubation with anti-tubulin primary antibody (clone B-5-1-2; Sigma) diluted in 5% BSA/PBS buffer for 2 h. After washes, cells were incubated with secondary antibody (Alexa Fluor 594 Phalloidin; Thermo Fisher Scientific) diluted in 5% BSA/PBS buffer for 1 h. After washes, slides were mounted using a mowiol mounting solution containing 4’,6’-diamino-2-phenylindole (DAPI) to counterstain the DNA. Slides were visualized at room temperature by using an inverted confocal microscope (LSM 780; Carl Zeiss).

## Results

### *RND1* transcripts are rapidly induced by CPT

To determine the RHO GTPases that are induced early in response to CPT, we treated human osteosarcoma U2OS cells for short times (1 h and 2 h), and analyzed RHO GTPase mRNA expression by reverse transcription followed by qPCR (RT-qPCR) (Fig. 1a). CPT efficiently induced TOP1cc in U2OS cells (Fig. 1b) as previously reported^38^. Among the RHO GTPase family, the two atypical members *RND1* and *RHOV*, were increased by CPT with *RND1* displaying an approximately 4 and 12 folds’ increase after 1 h and 2 h, respectively, and *RHOV* an approximately 3 and 4 folds’ increase (Fig. 1a). *RHOB* also increased under these conditions (Fig. 1a), as previously reported^9^. Among the two newly identified RHO GTPases, RND1 and RHOV, which are induced early by CPT (Fig. 1a), we further characterized RND1.

Kinetics of *RND1* mRNA induction in U2OS cells showed that at 25 µM of CPT, *RND1* increased within 1 h and reached a plateau after 2 h (Fig. 1c). To investigate whether the induction of *RND1* mRNA was dose-dependent, cells were treated for 2 h with increasing CPT concentrations. *RND1* induction was detected at 1 µM and increased with increasing concentrations of CPT (Fig. 1d). *RND1* induction was also observed in other human cell lines (glioblastoma U87, colon carcinoma HCT116, primary lung WI38 hTERT), and mouse cell lines (melanoma B16F10, embryonic NIH3T3) treated with CPT (Fig. 1e). Under these conditions, CPT efficiently induced TOP1cc in all these cell lines (Fig. 1f). Meta-analysis of microarray databases further supports the increase of *RND1* mRNA levels after short treatment with CPT or its water-soluble derivatives, topotecan and irinotecan, in human and rodent cell lines, and in tissues (Supplementary Figure 1A). In contrast, the two RND1 homologs, RND2 and RND3 were not induced after a short treatment with CPT in the cell lines analyzed (Fig. 1a, Supplementary Figure 1B, C).

As other groups^39^, we could not find or generate high-affinity antibodies that react specifically with endogenous RND1. Therefore, to determine whether the increase in *RND1* transcript levels could be associated with an increase in RND1 protein levels in CPT-treated cells, we generated U2OS cells stably expressing low or high levels of V5-tagged *RND1* transcripts (Fig. 1g). Cells with low and high levels of *RND1* transcripts (Fig. 1g) expressed low and high levels of RND1-V5 protein (Fig. 1h), respectively, suggesting that increasing *RND1* transcript expression also increases RND1 protein levels. Altogether, these results identify *RND1* as a new early-inducible RHO GTPase gene in response to CPT.

### *RND1* transcripts are closely associated with the presence of TOP1cc

CPT has for sole cellular target the TOP1cc^14^. To assess whether TOP1cc stabilization by CPT primes the increase of *RND1* mRNA levels, we examined whether other agents that induce TOP1cc would also induce *RND1*. Oxidative- and UV-mediated DNA lesions give rise to elevated levels of TOP1cc (see Table 1 in ref. ^13^). As a result, H_2_O_2_ and UV light induce cellular TOP1cc (Supplementary Figure 2A)^18,40^. Figure 2a shows that both agents increased *RND1* mRNA levels. Conversely, agents that do not induce TOP1cc, including the hypoxia-mimicking agent cobalt (II) chloride (CoCl_2_), the dihydrofolate reductase inhibitor methotrexate, and the tubulin inhibitor paclitaxel, did not increase *RND1* (Fig. 2b), under conditions where they exert their expected biological effects (for CoCl_2_, see Supplementary Figure 2b; for methotrexate, see Supplementary Figure 2c; for paclitaxel, see Supplementary Figure 2d).

Because CPT-induced TOP1cc are reversible^41^, we further examined *RND1* transcripts following CPT removal. After termination of the CPT treatment, *RND1* mRNA returned to their baseline levels (Fig. 2c, top panel) as TOP1cc reversed (Fig. 2c, bottom panel). Because the stabilization of TOP1cc decreases TOP1 activity leading to topological stress^14^, we have examined *RND1* induction in TOP1-depleted cells. Figure 2d shows that siRNA-mediated depletion of TOP1 in U2OS and WI38 hTERT cells did not increase *RND1* mRNA levels, suggesting that TOP1cc rather than inhibition of TOP1 activity promote *RND1* induction. Collectively, these results indicate that the increase of *RND1* transcripts is closely associated with the presence of TOP1cc.

### CPT increases *RND1* transcription and *RND1* transcript stability

**Fig. 3:**
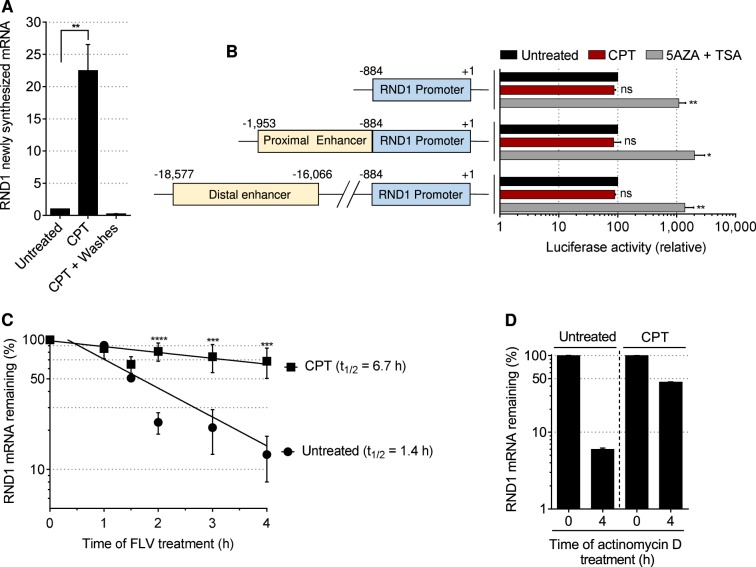
CPT increases *RND1* transcription and *RND1* transcript stability. **a** U2OS cells were treated with 25 µM CPT for 1 h and washed and cultured in CPT-free medium (CPT + Washes) for 0.5 h to allow reversion of TOP1cc. At the end of each time point, 0.2 mM EU was added to the culture medium for 2 h, after which EU-labeled nascent RNAs were captured. Nascent *RND1* RNAs were then analyzed by RT-qPCR (means ± SEM, *n* = 3; except for the “CPT + Washes” time point, for which *n* = 2). **P < 0.01 by unpaired *t* test. **b** Left panel: Diagram of pGL3-*RND1* promoter constructs (minimal promoter alone, minimal promoter with a proximal or a distal enhancer in order of appearance). Right panel: Luciferase activity of U2OS cells treated with 25 µM CPT for 2 h (minimal and distal promoter) or 3 h (proximal promoter). For the positive control, luciferase activity of U2OS cells treated with 1 µM 5-aza-2’-deoxycytidine (5AZA) for 72 h and with 100 nM trichostatin A (TSA) for 24 h (means ± SD, *n* = 3), *P < 0.05, **P < 0.01 by one-way ANOVA. **c** U2OS cells were left untreated or were treated with 25 µM CPT for 2 h before the addition of the transcription inhibitor flavopiridol (FLV, 1 µM) for the indicated times. *RND1* mRNA was then analyzed by RT-qPCR and normalized to the level at the time of FLV addition (means ± SD, *n* = 3). ***P < 0.001, ****P < 0.0001, by two-way ANOVA. The half-life (*t*_1/2_) of *RND1* mRNA is indicated. **d** Experiments were performed as in (**c**) with the transcription inhibitor actinomycin D (10 µg/ml). A representative experiment out of two is shown (means ± SD of triplicate samples). Ns, not significant.

The early increase of *RND1* mRNA in CPT-treated cells could depend on an increase in transcription and/or in transcript stability. Analysis of *RND1* transcription by capture of nascent transcripts followed by RT-qPCR showed that CPT increased by approximately 20 folds the transcription of *RND1* gene (Fig. 3a). This increase fully reversed after the removal of CPT (Fig. 3a), indicating that *RND1* transcription is closely related to the presence of TOP1cc. To determine whether the increase of *RND1* transcription in CPT-treated cells would depend on the activity of its promoter, we measured the activity of a luciferase reporter gene placed under the control of the *RND1* minimal promoter either alone or together with a proximal or a distal enhancer region^42,43^. Fig. 3b shows that CPT did not increase luciferase activity in cells transfected with each of these constructs, suggesting that the increase in *RND1* transcription by CPT might not primarily depend on an increased activity of its minimal promoter and the tested enhancers.

Next, we compared the stability of *RND1* mRNA between untreated and CPT-treated cells. Experiments performed in the presence of the transcription inhibitor flavopiridol showed that the half-life of *RND1* mRNA was greatly prolonged in CPT-treated cells (Fig. 3c). Similar results were obtained with the transcription inhibitor actinomycin D (Fig. 3d). Upon exposure to CPT, TOP1 has been reported to be degraded over time in a transcription-dependent manner^22,44,45^. Hence, the use of transcription inhibitors to analyze the lifespan of *RND1* mRNA in CPT-treated cells is likely to sustain the levels of TOP1cc during the time course of these experiments, which might contribute to further increase the half-life of *RND1* mRNA. Altogether, these data indicate that the increase of *RND1* transcript levels in response to CPT is associated with an increase in both *RND1* transcription and *RND1* transcript stability.

### PARP-1 increases *RND1* transcription in response to TOP1cc

PARP-1 can promote gene transcription^46–49^ and transcript stability^50^ via the addition of poly(ADP-ribose) residues (PAR) on proteins, an activity named PARylation. Because a short time CPT treatment increases PARP-1 activity^31^, we examined whether PARP-1 could promote the increase in *RND1* transcript levels in CPT-treated cells.

**Fig. 4:**
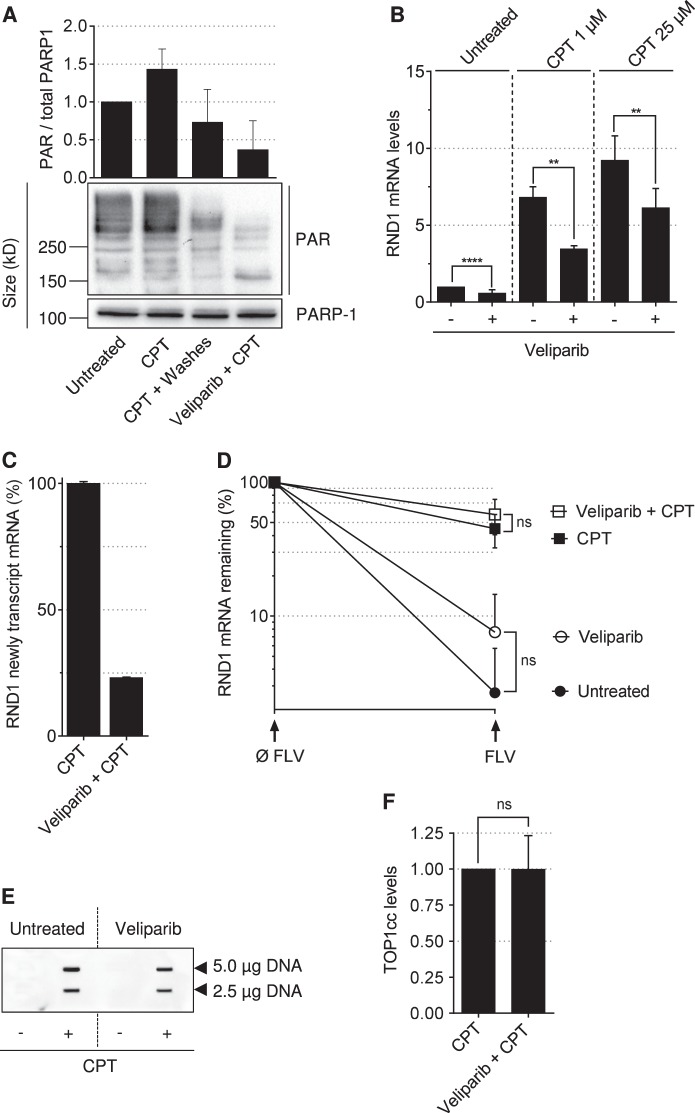
The PARP-1 inhibitor veliparib prevents CPT-induced *RND1* transcription. **a** U2OS cells were treated with 25 µM CPT for 2 h and washed and cultured in CPT-free medium (CPT + Washes) for 2 h to allow reversion of TOP1cc. When indicated, cells were pretreated with 5 µM veliparib for 1 h. The expression of PAR and PARP-1 were analyzed by Western blotting. The top panel shows quantification of PAR normalized to PARP-1 (means ± SD, *n* = 2). **b** RT-qPCR analysis of *RND1* mRNA in U2OS cells treated with 5 µM veliparib for 1 h before the addition of CPT for 2 h (means ± SD, *n* ≥ 3). **P < 0.01, ****P < 0.0001 by unpaired *t* test. **c** U2OS cells were treated with 5 µM of veliparib for 1 h before the addition of 25 µM CPT for 2 h. At the end of each time point, 0.2 mM EU was added to the culture medium for 2 h, after which EU-labeled nascent RNAs were captured. Nascent *RND1* RNAs were then analyzed by RT-qPCR. Data were normalized to the level of CPT-treated cells, which was taken at 100%. A representative experiment out of two is shown (means ± SD of triplicate samples). **d** U2OS cells were treated with veliparib (5 µM, 1 h) followed by the addition of CPT (25 µM, 1 h). After which, the transcription inhibitor FLV (1 µM) was added for 4 h. *RND1* mRNA expression was analyzed by RT-qPCR and normalized to the level at the time of FLV addition, which was set to 100 % (means ± SD, *n* = 3). Ns, not significant by two-way ANOVA. **e**, **f** U2OS cells were treated with 5 µM veliparib for 1 h before the addition of 25 µM CPT for 2 h, and TOP1cc were detected by probing two concentrations of genomic DNA (5 and 2.5 µg) with an anti-TOP1cc antibody. **e** Representative experiment. **f** Quantification of TOP1cc in veliparib + CPT-treated cells normalized to values from CPT-treated cells (means ± SEM, *n* = 3). Ns, not significant by unpaired *t* test.

As reported^31^, CPT increased protein PARylation (Fig. 4a). Protein PARylation was reversible and returned to its baseline level after CPT removal (Fig. 4a), a similar effect to that of *RND1* mRNA levels (Fig. 2c) and *RND1* gene transcription (Fig. 3a). Then, we assessed whether inhibiting PARP-1 activity would prevent the induction of *RND1* mRNA. The PARP-1 inhibitor veliparib partially prevented the induction of *RND1* mRNA in response to CPT (Fig. 4b) under conditions where it prevented protein PARylation (Fig. 4a). Then, we asked whether PARP inhibition would decrease *RND1* transcription and/or *RND1* transcript stability. In CPT-treated cells, veliparib strongly inhibited *RND1* transcription (Fig. 4c), while it did not decrease the half-life of *RND1* transcripts (Fig. 4d). As previously reported^38^, veliparib did not affect TOP1cc levels in response to CPT (Fig. 4e, f), which further suggests that PARP-1 is downstream from TOP1cc to increase *RND1* transcription. Altogether, these results suggest that, in CPT-treated cells, TOP1cc stabilization increases PARP-1 activity, which in turn increases the transcription of *RND1* gene, leading to an increase of *RND1* transcripts.

### RND1 increases PARP-1 expression in a positive feedback loop

**Fig. 5:**
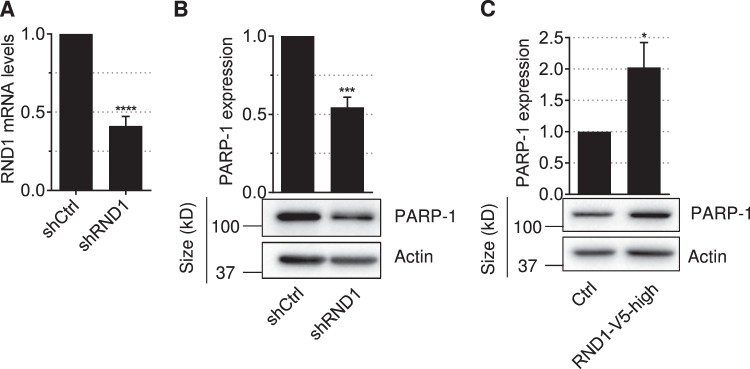
RND1 expression regulates PARP-1 expression. **a**, **b** U2OS cells were stably expressing shRNAs against RND1 (shRND1) or against a control sequence (shCtrl). **a** RT-qPCR analysis of *RND1* mRNA (means ± SD, *n* = 4). ****P < 0.0001 by unpaired *t* test. **b** Western blotting analysis of PARP-1. The top panel shows quantification of PARP-1 normalized to actin or tubulin (means ± SD, *n* = 5). ***P < 0.001 by unpaired *t* test. **c** Western blotting analysis of PARP-1 in U2OS stably expressing high levels of V5-tagged RND1 (RND1-V5-high, see panel Fig. 1g, h) or EGFP (ctrl). The top panel shows quantification of PARP-1 normalized to actin (means ± SD, *n* = 3). *P < 0.05 by unpaired *t* test.

Next, we considered whether there is a cross-talk between PARP-1 and RND1 or whether the talk is limited to one direction in which PARP-1 induces RND1. To test this, we asked whether modulating RND1 expression would modulate PARP-1 expression. Downregulation of *RND1* mRNA levels by shRNA in U2OS cells (Fig. 5a), decreased PARP-1 expression (Fig. 5b). Conversely, U2OS cells overexpressing RND1 (characterized in Fig. 1g, h), also overexpressed PARP-1 (Fig. 5c). These results suggest that PARP-1 activity increases RND1, which in turn increases PARP-1 expression in a positive feedback loop.

### DNA-PK-dependent DSB signaling prevents the induction of *RND1* transcripts by CPT

**Fig. 6:**
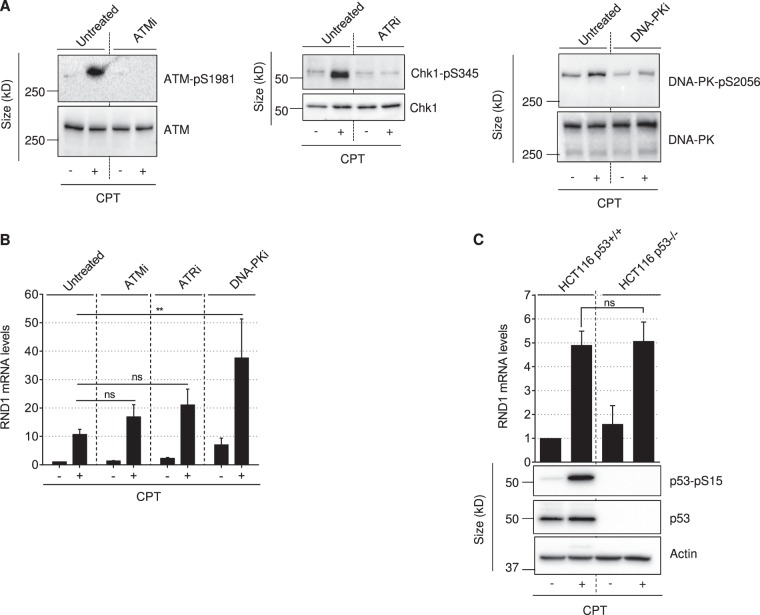
DNA-PK inhibition increases the induction of *RND1* transcripts by CPT. **a**, **b** U2OS cells were treated with ATMi (10 µM), ATRi (10 µM) or DNA-PKi (10 µM) for 1 h before the addition of 25 µM CPT for 2 h. **a** Western blotting analysis of the indicated proteins. **b** RT-qPCR analysis of *RND1* mRNA (means ± SEM, *n* ≥ 3), **P < 0.01, by one-way ANOVA. **c** HCT116 *p53*+/+ and HCT116 *p53*−/− cells were treated with 25 µM CPT for 2 h. Top panel: *RND1* mRNA was analyzed by RT-qPCR (means ± SEM, *n* = 3). Ns, not significant by one-way ANOVA. Bottom panel: Western blotting analysis of p53-pS15 and p53. Actin: loading control. Ns, not significant.

Because CPT-induced TOP1cc can lead to the production of DSBs^19–21,22–24^, we examined the role of DSB signaling in the induction of *RND1*. ATM, ATR and DNA-PK are serine/threonine kinases that are readily activated by DSBs, and phosphorylate various DNA damage response proteins such as p53^51^. Consistent with that, CPT activated these three kinases in U2OS cells as demonstrated by autophosphorylation of ATM at S1981 (ATM-pS1981), phosphorylation of the ATR substrate Chk1 at S345 (Chk1-pS345), and autophosphorylation of DNA-PK at S2056 (Fig. 6a). To determine their potential role in *RND1* induction, we assessed whether *RND1* induction is modified by specific chemical inhibitors of these kinases in CPT-treated U2OS cells: the ATM inhibitor (ATMi) KU55933, the ATR inhibitor (ATRi) VE-821 and the DNA-PK inhibitor (DNA-PKi) NU7441 (Fig. 6a). Figure 6b shows that DNA-PKi, and in a lesser extend ATMi and ATRi, increased the induction of *RND1* mRNA in response to CPT. Because p53 is phosphorylated and activated by these kinases^51^, we examined *RND1* induction in *p53*+/+ and *p53*−/− HCT116 cells exposed to CPT. As expected, CPT induced p53 phosphorylation at S15 and increased p53 protein level in *p53*+/+ HCT116 cells (Fig. 6c, bottom panels). We found that *p53*+/+ and *p53−/−* HCT116 cells both displayed similar induction of *RND1 mRNA* in response to CPT (Fig. 6c, top panel). Collectively, our experiments suggest that the DNA-PK-dependent DSB signaling prevents the induction of *RND1* transcripts by CPT. However, this DSB signaling pathway is not mediated by p53.

### RND1 reduces the sensitivity of cells exposed to CPT

**Fig. 7:**
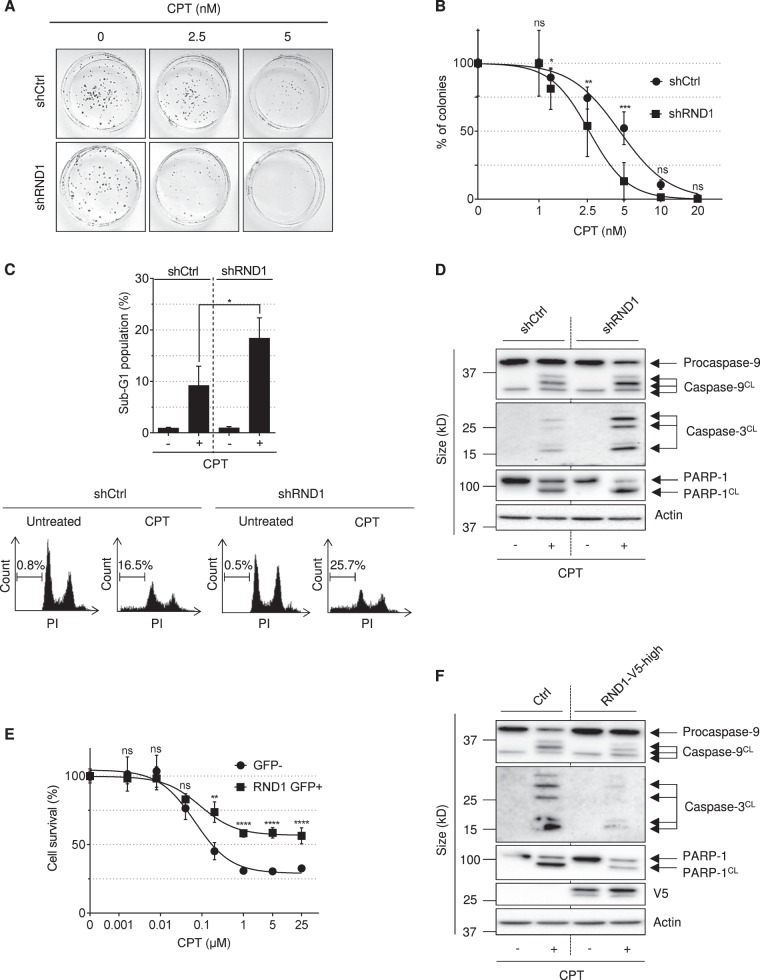
RND1 protects cells against CPT-induced apoptosis. **a**, **b** Colony formation assay in U2OS cells stably expressing shRNAs against RND1 (shRND1) or against a control sequence (shCtrl), and treated with increasing concentrations of CPT (from 1.25 to 20 nM). Percentages of colonies were assessed after 10 days by counting the number of colonies and normalized to that of untreated cells, which was set at 100% (means ± SD, *n* = 4), *P < 0.05, **P < 0.01, ***P < 0.001, by two-way ANOVA. **c** U2OS shRND1 or shCtrl cells were treated with 25 µM CPT for 24 h. Percentage of sub-G1 cell population was analyzed by flow cytometry. The top panel shows quantification of sub-G1 cell population (means ± SEM, *n*=3). *P < 0.05 by unpaired *t* test. Bottom: one representative experiment is shown. **d** Western blotting analysis of the indicated proteins in U2OS shRND1 or shCtrl cells treated with 25 µM CPT for 24 h. Caspase-9^CL^: cleaved caspase-9, Caspase-3^CL^: cleaved caspase-3, PARP-1^CL^: cleaved PARP-1. Data shown are representatives from three experiments. (**e**) U2OS cells were transfected with pEGFP-RND1 plasmid. Forty-eight hours after transfection, GFP- and GFP + U2OS cells were separated by cell sorting. GFP− and RND1 GFP+ U2OS cells were treated with increasing concentrations of CPT (from 0.0016 µM to 25 µM). Seventy-two hours after treatment, cell survival was analyzed by a WST-1 assay. A representative experiment out of three is shown (means ± SD for triplicate samples). Ns not significant, **P < 0.01, ****P < 0.0001, by two-way ANOVA. **f** Similar experiments as in panel (**d**) in U2OS Ctrl and RND1-V5-high cells. Ns not significant

To assess the potential role of RND1 in the cellular response to TOP1cc stabilization, we first compared the sensitivity of shCtrl and shRND1 U2OS cells (characterized in Fig. 5a) to CPT treatment. Cells were treated with increasing concentrations of CPT, and CPT sensitivity was assessed by clonogenic assays. Figure 7a, b shows that shRND1 cells formed significantly less clones in response to CPT than shCtrl cells. The increased sensitivity of shRND1 cells to CPT was further associated with an increase of apoptotic marks such as sub-G1 population (Fig. 7c), and the cleavage of caspase-9, caspase-3 and PARP-1 (Fig. 7d). Conversely, overexpression of RND1 in U2OS cells (Supplementary Figure 3) decreased cell sensitivity to CPT as measured by WST-1 survival assays (Fig. 7e) and decreased apoptotic marks (Fig. 7f). Together, these results demonstrate that RND1 protects cells against CPT, likely by preventing apoptosis.

## Discussion

**Fig. 8:**
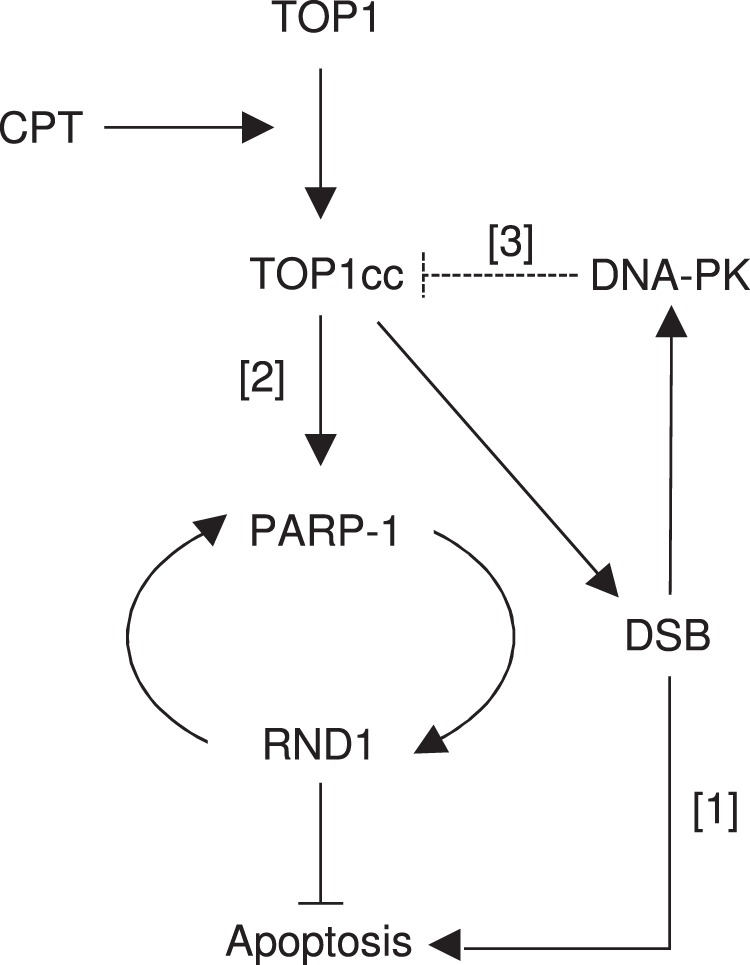
Proposed model for the induction of RND1 in response to CPT. CPT stabilizes TOP1cc, which in turn induce DSBs and apoptosis^25^ [1]. TOP1cc also activates a PARP-1-RND1 pathway that counteracts the induction of apoptosis [2]. DNA-PK-dependent DSB signaling prevents RND1 induction, possibly by promoting TOP1cc removal^22,52^ [3].

Here we identified RND1 as an early inducible RHO GTPase gene in response to CPT. This is the first time that an atypical RHO is reported to respond early to DNA damaging agents. Our data support a model depicted in Fig. 8 in which CPT-induced TOP1cc stabilization increases PARP-1 activity that triggers *RND1* transcription, which elevates the levels of *RND1* transcripts (and likely also the protein). In turn, the increase of RND1 protein levels promotes an increase of PARP-1 protein levels, suggesting a positive feedback loop between PARP-1 and RND1 in response to CPT. The increase of RND1 induced by the TOP1cc-PARP-1 pathway protects cells against CPT, likely by inhibiting apoptosis. PARP-1-independent pathways probably also contribute to the increase of *RND1* transcript levels, as the inhibition of PARP-1 activity with veliparib does not completely suppress CPT-induced *RND1* transcripts. Such pathways might involve an increased stability of *RND1* transcripts as our analysis shows that CPT extends the half-life of *RND1* mRNA in a PARP-1-independent manner. CPT-induced TOP1cc also induce DSBs, which activate DNA-PK that reduces the induction of *RND1*. DNA-PK could reduce the induction of *RND1* by promoting TOP1 proteolysis as previously reported^22,52^. Albeit in a lesser extent, ATM, which can activate DNA-PK^22^, also reduces the induction of *RND1*. Consistent with that, ATM has also been reported to promote TOP1 proteolysis^53,54^.

Our study uncovers the close relationship between TOP1cc and the transcription of *RND1*. Indeed, CPT, which induces *RND1* transcription, has for sole cellular target the TOP1cc^14^, and reversion of TOP1cc following termination of the CPT treatment readily restores the baseline levels of *RND1* transcription and *RND1* transcripts. In addition,  H_2_O_2_ and UV light, which induce DNA lesions that interfere with TOP1 nicking-closing activity and give rise to elevated levels of TOP1cc^15–18,40^, also increase *RND1* transcript levels. Besides TOP1 inhibitors and DNA alterations (see Table 1 in ref. ^13^), several other processes lead to persistent TOP1cc, including ribonucleotide incorporation into DNA^55–57^, genetic defects such as ATM defect^53,54^, and transcriptional activation^58^. Hence, the increased transcription of *RND1* due to TOP1cc stabilization might be a frequent event occurring under both physiological and stress conditions.

An early response to CPT is the global inhibition of transcription^22,45^. However, genes are differentially affected by CPT and a fraction of them, primarily the short and low-expressed genes, are upregulated^27–29^. In accordance with this, *RND1* is a short gene (8.7 Kbp), with a low-expression in most healthy tissues apart from brain and liver^2^ and in addition, *RND1* expression is significantly downregulated in several aggressive tumors compared to normal tissues^39,59,60^. The mechanisms by which CPT enhances transcription of some genes are largely unknown. Here we reported that CPT induces PARP-1 activity, which in turn stimulates *RND1* transcription. This effect is likely related to TOP1cc. Similar to CPT, H_2_O_2_ and UV light induce persistent TOP1cc^18,40^, increase *RND1* transcript levels (this study), and also increase PARP-1 activity^61,62^. Whether TOP1cc-induced PARP-1 activity is a common mechanism for CPT to promote gene transcription or whether it is restricted to *RND1* gene remains to be investigated.

It is now well documented that PARP-1 regulates transcription^63^ asides from its well-recognized role in DNA repair^64^. PARP-1 is enriched to the promoters of actively transcribed genes^65^ and, stimulates transcription initiation by maintaining an ‘open’ chromatin environment through PARylation of core histones and exclusion of histone H1 from the DNA^48,65^, or inhibition of histone H3K4me demethylation by KDM5B^47^. PARP-1 could also promote transcription by stimulating transcription elongation. PARP-1 PARylates subunits of the negative elongation factors (NELF), NELF-A and NELF-E, which triggers the release of RNA polymerase II from its paused site for productive elongation^49^. Our results showing that increased *RND1* transcription by CPT does not relies on increased activity of its promoter suggest that PARP-1 might primarily function in stimulating transcription elongation of *RND1* gene. PARP-1-independent pathways probably also contribute to the increase of *RND1* transcription, as PARP-1 inhibition does not completely suppress CPT-induced *RND1* transcription. A previous study shows that the CPT derivatives topotecan can stimulate *UBE3A* transcription by downregulating the expression of its antisense transcript^66^. The non-coding RNA AGAP2-AS1 has been reported to inhibit *RND1* transcription^67,68^, which raises the possibility that CPT could inhibit AGAP2-AS1 transcription, which in turn could increase *RND1* transcription.

Lastly, our analysis shows that RND1 protects cells against CPT-induced apoptosis and hence favors cell resistance. These findings extend the role of RND1 beyond its original function in the disassembly of actin filament structures and loss of cell adhesion^2^ as well as in embryonic development, where it promotes the formation and maturation of neuronal protrusions^69,70^ and controls gastrulation movements^71^. In addition, RND1 behaves as a tumor suppressor gene. *RND1* expression levels decrease in several aggressive tumors^39,59,60^, and RND1 loss in immortalized mammary cells can initiate breast tumorigenesis and promotes metastasis^39^. Even in tumor cell lines expressing low levels of RND1 such as MCF-7 cells^39^, U87 cells^59^ and U2OS cells, RND1 could be transiently induced by TOP1cc to resist to CPT derivatives. This potential selective advantage of tumor cells suggests that inhibiting RND1-dependent signaling could sensitize them to CPT derivatives.

## Electronic supplementary material

Supplementary Data
