# Supplementary material for: PARP-1-dependent RND1 transcription induced by topoisomerase I cleavage complexes confers cellular resistance to camptothecin
Source: Cell Death Dis. 2018 Sep 12;9(9):931. doi: 10.1038/s41419-018-0981-3 (PMC6135836; doi:10.1038/s41419-018-0981-3)

# Supplementary Data

# PARP-1-dependent RND1 transcription induced by topoisomerase I cleavage complexes confers cellular resistance to camptothecin

Laetitia Mouly, Kenza Mamouni, Remi Gence, Agnese Cristini, Julia Cherier, Adrien Castinel, Morgane Legrand, Gilles Favre, Olivier Sordet, Sylvie Monferran

---

## SUPPLEMENTARY FIGURE LEGENDS

**Supplementary Figure 1**

(**A**) Meta-analysis of *RND1* expression fold change after treatment with CPT or its derivatives irinotecan or topotecan in human cancer cells (OCI-LY3; MCF-7; PC3; HCT116) or rat tissue (bone marrow) from gene expression datasets (at least $p<0.05$ by unpaired *t* test). Fold induction equals a value of 1.4 to 2 (+) or of 2.1 to 6 (++). (**B** and **C**) The expression of *RND2* (**B**) and *RND3* (**C**) was analyzed by RT-qPCR in U87 and WI38 hTERT cells treated with 25 $\mu$M CPT for 2 h (means ± SD, n = 3). Ns = not significant, **P<0.01, ****P<0.0001 by unpaired *t* test.

**Supplementary Figure 2**

(**A**) Detection of TOP1cc in U2OS cells treated with 25 mM H2O2 for 30 min. 10 µg of genomic DNA was probed with an anti-TOP1cc antibody. (**B**) Western blotting of HIF1α and actin in U2OS cells treated for 2 h with 100 µM CoCl2. (**C**) Cell survival of U2OS cells treated for 72 hours with 50 µM methotrexate, normalized to the level of untreated cells which was set at 100%. (**D**) Representative images of U2OS cells treated with 10 $\mu$M paclitaxel for 2 h, fixed and then stained for tubulin (red). DNA was counterstained with DAPI (blue).

**Supplementary Figure 3**

U2OS cells were transfected with pEGFP-RND1 plasmid. 48 hours after transfection, GFP- and GFP+ U2OS cells were separated by cell sorting in comparison with signals observed in non-transfected cells.

# Supplemental Figure 1

**A**

| **Sample** | **Treatment** | **Time of treatment** | **Induction of RND1 mRNA** |
|---|---|---|---|
| Bone marrow (r) | Topotecan | 30 min | + |
| OCI-LY3 (h) | Irinotecan | 6 h | ++ |
| MCF-7 (h) | Irinotecan | 6 h | + |
| PC3 (h) | CPT | 6 h | ++ |
| HCT116 (h) | CPT | 2 h | ++ |

h: human; r: rodent

**B**

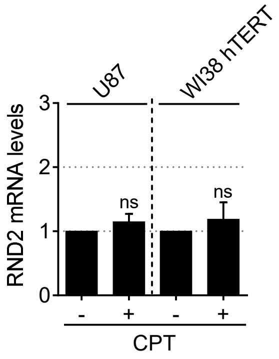


**C**

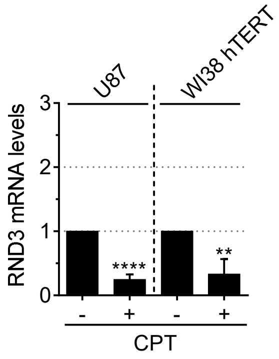

# Supplemental Figure 2

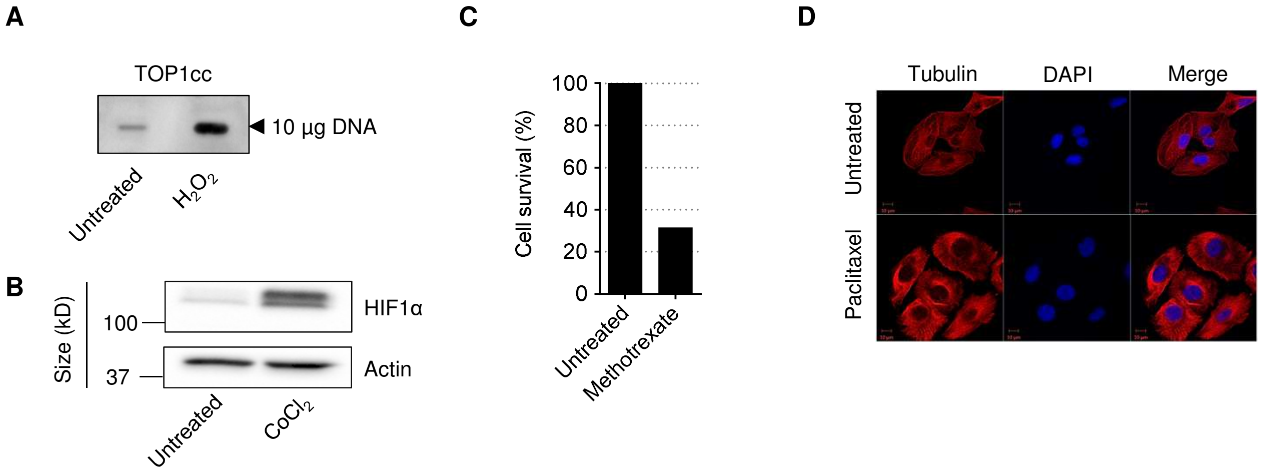

# Supplemental Figure 3

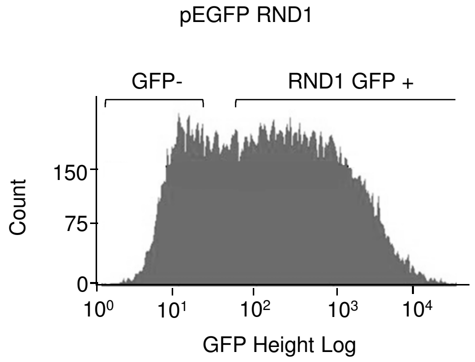

Supplement: Supplementary file 1 — Supplementary Data [file 41419_2018_981_MOESM1_ESM.pdf]
